# Detection of EGFR mutation of pulmonary adenocarcinoma in sputum using droplet digital PCR

**DOI:** 10.1186/s12890-021-01468-9

**Published:** 2021-03-23

**Authors:** Tetsuya Isaka, Tomoyuki Yokose, Hiroyuki Ito, Haruhiko Nakayama, Yohei Miyagi, Haruhiro Saito, Munetaka Masuda

**Affiliations:** 1 Department of Thoracic Surgery, Kanagawa Cancer Center, 2-3-2 Nakao, Asahi, Yokohama, Kanagawa 241-8515 Japan; 2 Department of Surgery, Yokohama City University, 3-9 Fukuura, Kanazawa, Yokohama, Kanagawa 236-0004 Japan; 3 Department of Pathology, Kanagawa Cancer Center, 2-3-2 Nakao, Asahi, Yokohama, Kanagawa 241-8515 Japan; 4 Molecular Pathology and Genetics Division, Kanagawa Cancer Center Research Institute, 2-3-2 Nakao, Asahi, Yokohama, Kanagawa 241-8515 Japan; 5 Department of Thoracic Oncology, Kanagawa Cancer Center, 2-3-2 Nakao, Asahi, Yokohama, Kanagawa 241-8515 Japan

**Keywords:** Lung cancer, Adenocarcinoma, EGFR, Digital droplet PCR, Sputum, Cytology, STAS

## Abstract

**Background:**

It is still unclear whether epidermal growth factor receptor (EGFR) mutation of primary lung adenocarcinoma can be detected on sputum samples. This study aimed to examine EGFR mutations of primary lung adenocarcinoma in sputum samples using droplet digital polymerase chain reaction (ddPCR) and compare it with an EGFR mutation in surgically resected lung cancer.

**Methods:**

Sputum was prospectively collected from the patients before complete resection of the primary lung cancer at Kanagawa Cancer Center from September 2014 to May 2016. ddPCR was performed to detect EGFR exon 21 L858R point mutation (Ex21) and EGFR exon 19 deletion mutation (Ex19) in sputum samples from patients with lung adenocarcinoma. The concordance of EGFR mutation status in sputum samples and tumors in surgically resected specimen was evaluated for each positive and negative cytology group.

**Results:**

One hundred and eighteen patients with primary lung adenocarcinoma provided sputum samples. Sputum cytology was positive in 13 patients (11.0%). ddPCR detected two cases of Ex21 and two cases of Ex19 in sputum cytology positive cases. Compared to surgically resected specimens, the sensitivity, specificity, and positive predictive value of EGFR mutation (Ex19 and Ex21) detection were 80.0%, 100%, and 100%, respectively, in sputum cytology positive cases. In contrast, the sensitivity, specificity, and positive predictive value of EGFR mutation (Ex19 and Ex21) detection were 3.1%, 100%, and 100%, respectively, in sputum cytology negative cases.

**Conclusions:**

EGFR mutations in primary lung adenocarcinoma can be detected with high sensitivity in sputum samples if sputum cytology is positive.

## Introduction

Patients with advanced non-small cell lung cancer (NSCLC) harboring epidermal growth factor receptor (EGFR) mutations can experience improved prognosis when treated with EGFR-tyrosine kinase inhibitor [1, 2]. EGFR-mutated lung cancer is reported to comprise approximately 45% of the NSCLC cases in Asia and approximately 15% in Europe and the United States [3–6]. Approximately 90% of EGFR mutations are either EGFR exon 21 L858R point mutations (Ex21) or EGFR exon 19 deletion mutations (Ex19) [7]. Detection of EGFR mutations is the first process in deciding the treatment for patients with advanced NSCLC. Currently, bronchoscopic biopsy, computed tomography (CT)-guided biopsy, and surgical biopsy are performed to collect tumor samples. However, these methodologies have various complications and are invasive for patients, which can be especially problematic for those in poor condition [8–11]. In addition to these biopsies, non-invasive liquid biopsy using blood (liquid biopsy) has recently been performed to detect EGFR mutations. However, a meta-analysis reported that the sensitivity and specificity of the detection of EGFR mutations in blood were 65% and 91%, respectively, with recurring low detection sensitivity [12]. Development of a methodology capable of more safely collecting tumor samples and more accurately detecting EGFR mutations in patients with lung cancer is needed.

Sputum can be collected non-invasively from patients. Whether EGFR mutation analysis can be performed accurately using sputum is unclear. The sensitivity of detecting lung cancer in sputum cytology is reported to be approximately 40 to 66% [13, 14]. However, sputum has been considered to be unsuitable for EGFR mutation analysis because it contains many normal cells, such as bronchial epithelium and inflammatory cells, with tumor cells comprising < 1% of the total number of cells in sputum [15]. Furthermore, primary lung adenocarcinoma, for which molecular analysis is especially important in deciding the treatment protocol, was reported to be less likely to be detected via sputum cytology compared to central squamous cell carcinoma [16]. Therefore, an ultrasensitive method for detecting tumor EGFR mutations in sputum sample is necessary. Furthermore, it is necessary to know the clinical characteristics of patients whose sputum contains a sufficient amount of tumor cells for the EGFR gene detection.

Droplet digital polymerase chain reaction (ddPCR) is a technology that can detect and quantify specific sequences with much higher sensitivity and specificity than conventional real-time PCR [17, 18]. ddPCR is based on the limiting dilution of DNA sample in microcompartments within droplets of a water-in-oil emulsion [17]. The sensitivity of ddPCR was reported to be approximately 0.03%-0.001% mutant DNA in the detection of EGFR T790M gene mutation and 0.0005% mutant DNA in the detection of KRAS gene mutation [19, 20]. Considering that it is difficult to detect ≤ 1% mutant DNA using conventional PCR [21, 22], ultrasensitive ddPCR is necessary to detect EGFR mutations in the tumor cells in sputum, because of the abundant normal cells or non-mutated DNA in the background of a sputum sample. However, to date, few reports have described the use of ddPCR in EGFR mutation analysis in sputum samples.

This study aimed to examine EGFR mutations of primary lung adenocarcinoma in sputum samples using ddPCR and compare it with an EGFR mutation in surgically resected lung cancer. This study also analyzed the clinicopathological features of patients in which sufficient tumor cells were detected in sputum for EGFR mutation testing via ddPCR.

## Materials and methods

### Patients and sputum collection

Sputum was prospectively collected from the patients before complete resection of the primary lung cancer at Kanagawa Cancer Center from September 2014 to May 2016. Patients with pure ground glass nodule, synchronous multiple lung cancer, and uncontrollable other cancer were excluded from this study. Patients undergoing preoperative radiation therapy or chemotherapy, and bronchoscopy within one week were also excluded. All patients provided informed consent. This study was performed in accordance with relevant guideline and was approved by the Kanagawa Cancer Center institutional review board (25 Ken—64 and 2019 Eki-14).

Each patient was given a container with YM fixative solution (50% ethyl alcohol and 2% polyethylene) for collection of sputum for 3 days before surgery [23]. Patients were instructed to collect early morning sputum just after gargling and to shake the container approximately 20 times after each sputum collection so that the sputum mixed with the fixative. The patients were instructed to store the containers in a refrigerator.

### Cytology

After centrifugation, a total of two cytological specimens were prepared by rubbing the sputum sample between two glass slides. Each cytological specimen was stained with Papanicolaou stain after 95% ethyl alcohol fixation. Cytology was classified by an expert cytologist as follows: (1) Insufficient material; (2) Class I: absence of atypical or abnormal cells; (3) Class II: atypical cytology but no evidence of malignancy; (4) Class III: cytology suggestive of, but not conclusive for, malignancy; (5) Class IIIa: probably benign atypia; (6) Class IIIb: malignancy suspected; (7) Class IV: cytology strongly suggestive of malignancy; and (8) Class V: cytology conclusive for malignancy. Final diagnosis was made by a pathologist in cases of class III or higher. Sputum cytology positive [SC (+)] was defined as patients whose sputum cytology was class III or higher. Sputum cytology negative [SC (−)] was defined as patients whose sputum cytology was lower than class III, including cases with insufficient material. SNC (sputum not collected) was defined as patients who could not collect sputum because of a lack of sputum.

### DNA extraction and ddPCR

ddPCR was performed to detect Ex21 and Ex19 in patients with lung adenocarcinoma using the same sputum samples used for cytological examination. The cover glass was peeled off after immersing the glass slide with the cytological specimen in xylene. After applying 50% diluted Marinol in xylene, the Marinol was cured on the extender for 30 to 60 min. The sample was then immersed in Milli-Q^®^ water for approximately 15 min to soften the encapsulant, and the sheet-like cells were peeled off with a knife and placed in a tube. After dissolution and removal of the encapsulant, the sample was washed with alcohol and dried. DNA was extracted using the QIAamp DNA FFPE Tissue Kit (Qiagen, Inc., Valencia, CA, USA) according to the manufacturer's protocol. In all samples, the concentration of DNA was measured using Qubit^®^ (Life Technologies, Thermo Fisher Scientific, Carlsbad, CA, USA).

ddPCR was performed using the QX200 Droplet Digital PCR System (Bio-Rad, Hercules, CA, USA) according to the manufacturer's protocol. A total of 20 μL of ddPCR reaction mixture was prepared. The volume contained 10 μL of 2 × ddPCR Supermix for Probe (no dUTP) (Bio-Rad), 1 μL template DNA, 1.8 μL of forward and reverse primers (10 μM), 0.5 μL of FAM- and Hex-labeled probe, and 4.9 μL of nuclease-free water. A total of 20 μL/well of the sample solution was transferred to DG8 cartridges (Bio-Rad). After loading 70 μL/well of generator oil in the lower layer of the well, droplets were made using the Droplet Generator (Bio-Rad). After transferring 40 μL to each well of a PCR plate, the plate was sealed with a foil heat seal using a PX1 PCR Plate Sealer (Bio-Rad). PCR was performed using a C1000 Touch thermal cycler (Bio-Rad). The droplet generator partitions samples into approximately 20,000 droplets of identical volume. However, 12,000 to 16,000 droplets were finally used for the reaction because some droplets were lost in the transfer step.

EGFR p.L858R c.2573T>G (Bio-Rad, Catalog #10,049,550, Assay ID: dHsaMDV2010021) was used as the primer/probe mix to detect the Ex21 using ddPCR. The ddPCR EGFR Exon 19 Deletions Screening Kit (Bio-Rad, Catalog #12002392), which allows the quantification and screening of 15 EGFR exon 19 deletions, was used to detect Ex19. The negative template control contained reaction mixed with water, and the positive template control contained EGFR-mutated DNA (Ex21 and Ex19). QuantaSoft software (version 1.7.4) was used for the analysis. The presence or absence of EGFR mutations was determined by the threshold set automatically in the analysis mode of the software based on the criterion optimized in the analysis of each sample. If the automatic analysis did not work because of the small quantity of sample DNA, the threshold was set manually, based on the fluorescence amplitude of the positive control. ddPCR was performed multiple times to confirm reproducibility if the initial ddPCR result was positive for EGFR mutation. Moreover, the sputum sample was determined to be an EGFR mutant if the EGFR gene mutation detection rate was above the detection limit validated using each reagent. The limit of detection of EGFR p.L858R c.2573T>G to detect the Ex21 was 0.1% and that for the EGFR Exon 19 Deletions Screening Kit to detect the Ex19 was 0.5%.

### Pathological findings and EGFR mutation of surgically resected lung cancer specimens

The pathological diagnosis was made by an expert pathologist (Y.T.) based on hematoxylin and eosin (H&E) staining and Alcian blue staining of the tissue sections of formalin-fixed paraffin-embedded (FFPE) specimens. Elastica van Gieson staining was used to evaluate vascular and pleural invasion. Immunostaining of thyroid transcription factor-1 was performed if necessary. Spread through air space (STAS) was defined as the spread of lung cancer cells into air spaces in the lung parenchyma beyond the edge of the main tumor [24, 25]. The existence of STAS was evaluated based on H&E stains of FFPE sections of the tumor.

FFPE sections of the resected tumor were used to extract the DNA from the samples. Eight to ten sections (5–10 μm thick) where tumor diameter was maximum were used to extract DNA. The fragment method (sensitivity < 5% mutant DNA) was used to examine Ex19 [21]. The Cycleave method (sensitivity 1–5% mutant DNA) was used to examine Ex21 [21]. The concordance of EGFR mutation status detected in sputum samples and in surgically resected specimens was evaluated for SC (+) and SC (−) groups, respectively.

### Sub-analysis of risk factors of SC (+)

Sub-analysis of risk factors for SC (+) in patients with primary lung adenocarcinoma was performed because EGFR mutation cannot be detected if the sputum sample does not contain any malignant cells. The clinicopathological factors of SC (+) and SC (−) + SNC were compared between groups, and the risk factors for SC (+) were analyzed by multivariate analysis. Continuous and categorical variables between the two groups were compared using Mann–Whitney U test and Fisher's exact test, respectively. Receiver operating characteristics (ROC) curve analysis was performed to discriminate SC (+) from SC (−) + SNC via radiological examination (CT tumor size and positron emission tomography maximum standardized uptake value [PET SUVmax]). CT tumor size was defined as the maximum tumor diameter measured using high resolution CT (level 600 Hounsfield units [HU]; width 1600 HU) of 1 to 2 mm thickness. The preoperative PET-CT scan calculated the SUVmax of the tumor lesion where fluorodeoxyglucose F 18 (18F-FDG) accumulated. Logistic regression was performed to analyze the clinicopathological characteristics of patients regarding SC (+). Significance was defined as p < 0.05. Statistical analyzes were performed using EZR on R commander version 1.30 (Saitama Medical Center, Jichi Medical University, Saitama, Japan).

## Results

**Fig. 1:**
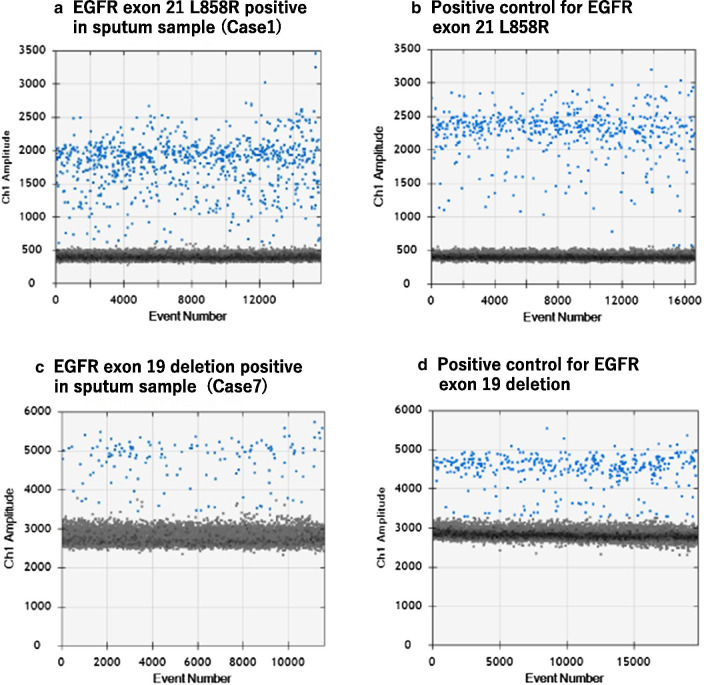
Droplet digital PCR data analysis. **a** 1-D plot with each droplet from a sample plotted on the graph of fluorescence intensity versus droplet number is shown. Blue dots denote EGFR mutant droplets and gray dots denote wild-type EGFR droplets. **a** Ex21 in sputum sample (Case1), **b** positive control for Ex21, **c** Ex19 in sputum sample (Case7), and **d** positive control for Ex19

Of the 118 patients who enrolled in this study, the number of patients with SC (+), SC (−), and NC was 13 (11.0%), 76 (64.4%), and 29 (24.6%), respectively. Table 1 summarizes the radiological and cytopathological findings, and the EGFR mutation status (sputum sample and surgical resected specimen) of 13 SC (+) cases. In addition, allel frequency in samples in which EGFR mutation were detected in sputum samples was also shown. Classes III, IIIa, IIIb, IV, and V included 2, 2, 3, 1, and 5 patients, respectively. STAS was detected in surgically resected specimens in 12 of 13 SC (+) cases. Ex21 and Ex19, and wild-type EGFR were observed in 2, 2, and 9 cases, respectively, based on ddPCR of the sputum. Among SC (+) samples, the EGFR mutation status of the main tumors in FFPE sections and in sputum were identical in 12 cases, and the sensitivity, specificity, and positive predictive value to detect EGFR mutations (Ex19 and Ex21) were 80.0%, 100%, and 100%, respectively (Table 2). In one discordant case (Case 4), the cytological examination of sputum was Class IIIa with suspected squamous cell carcinoma. Figure 1 shows the results of ddPCR data analysis (1-D plot). Ex21 (Case 1) and Ex19 (Case 7) were detected in sputum by ddPCR. The lowest allele frequency in the EGFR mutant SC (+) cases was 0.24% (Table 1).

**Table 1:** Radiological and cytopathological findings, and the EGFR mutation status (sputum sample and surgical resected specimen) of 13 sputum cytology positive cases

Patients	Tumor size (mm)	PET SUV max	Sputum cytology	Subtype of adenocarcinoma	STAS in permanent section	EGFR mutation in FFPE	EGFR mutation in ddPCR	Allele frequency (%)
Case 1	91	19.4	Class V (Ad)	Invasive papillary adenocarcinoma	(+)	Ex21	Ex21	19.4
Case 2	23	4.3	Class IIIb (Ad)	Invasive lepidic adenocarcinoma	(+)	Ex19	Ex19	0.6
Case 3	98	6.2	Class V (Ad)	Invasive mucinous adenocarcinoma	(+)	Wild	Wild	(−)
Case 4	32	3.0	Class IIIa (SQ)	Invasive lepidic adenocarcinoma	(+)	Ex21	Wild	(−)
Case 5	38	9.2	Class IIIa	Invasive mucinous adenocarcinoma	(+)	Wild	Wild	(−)
Case 6	29	7.8	Class III	Invasive acinar adenocarcinoma	(+)	Wild	Wild	(−)
Case 7	29	3.1	Class V (Ad)	Unknown	(−)	Ex19	Ex19	2.88
Case 8	37	8.7	Class IIIb	Invasive papillary adenocarcinoma	(+)	Ex21	Ex21	0.24
Case 9	41	6.9	Class III	Invasive solid adenocarcinoma	(+)	Wild	Wild	(−)
Case 10	21	7.4	Class V	Invasive acinar adenocarcinoma	(+)	Wild	Wild	(−)
Case 11	87	19.3	Class IIIb (Ad)	Invasive solid adenocarcinoma	(+)	Wild	Wild	(−)
Case 12	32	11.9	Class V (Ad)	Invasive papillary adenocarcinoma	(+)	Wild	Wild	(−)
Case 13	52	5.4	Class IV (Ad)	Invasive papillary adenocarcinoma	(+)	Wild	Wild	(−)

Ad, adenocarcinoma; SQ, squamous cell carcinoma; STAS, spread through air spaces; EGFR, epidermal growth factor receptor; ddPCR, droplet digital PCR; FFPE, formalin-fixed paraffin-embedded; Ex19, exon 19 deletion mutation; Ex21, exon 21 L858R point mutation

**Table 2:** Sensitivity, specificity, and positive predictive value of EGFR mutation (Ex19 and Ex21) detection in sputum cytology positive and negative cases

Sputum cytology positive cases	EGFR mutation (+) in surgical resected specimen	EGFR mutation (−) in surgical resected specimen	Total
EGFR mutation (+) in sputum sample	4	0	4
EGFR mutation (−) in sputum sample	1	8	9
Total	5	8	13
Sensitivity = 80.0% Specificity = 100% Positive predictive value = 100%			

Sputum cytology negative cases	EGFR mutation (+) in surgical resected specimen	EGFR mutation (−) in surgical resected specimen	Total
EGFR mutation (+) in sputum sample	1	0	1
EGFR mutation (−) in sputum sample	31	44	75
Total	32	44	76
Sensitivity = 3.1% Specificity = 100% Positive predictive value= 100%			

Ex19, exon 19 deletion mutation; Ex21, exon 21 L858R point mutation

**Fig. 2:**
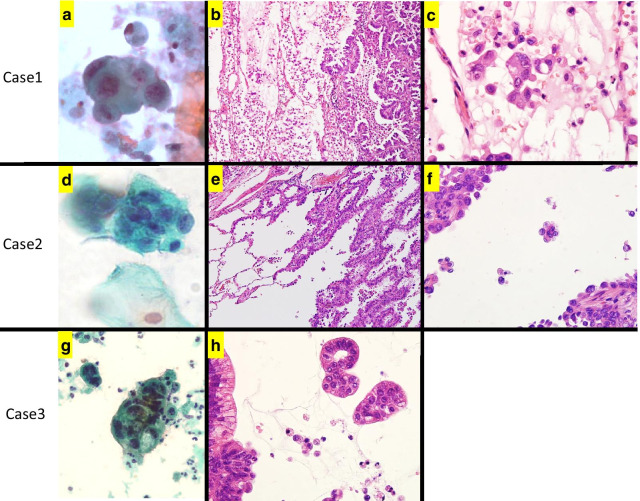
Cytological findings of sputum and histological findings of resected specimen in Case 1 to 3 of the 13 SC (+) cases. **a**–**c** Case 1 was diagnosed with invasive papillary adenocarcinoma with pT3N2M0 stage IIIA. **a** The sputum cytology revealed adenocarcinoma (class V). **b**, **c** STAS with solid nests or single cells features was detected in FFPE sections of the surgically resected tumors. **d–f** Case 2 was diagnosed with invasive lepidic adenocarcinoma with pT1cN1M0 stage IIB. **d** Adenocarcinoma was suspected (class IIIb) in the sputum cytology. **e**, **f** STAS with micropapillary feature was detected in FFPE sections of the surgically resected tumors. **g**, **h** Case 3 was diagnosed with invasive mucinous adenocarcinoma with pT3N2M0 stage IIIA. **g** The sputum cytology revealed adenocarcinoma (class V). **h** STAS with solid nests feature was detected in FFPE sections of the surgically resected tumors

Figure 2 presents the cytological findings of sputum and histological findings of resected specimen in Case 1 to 3 of the 13 SC (+) patients. In all three patients, the EGFR mutation status of the main tumors in FFPE sections and in sputum were identical. In all three patients, STAS was detected in FFPE sections of the surgically resected tumors.

Among 76 patients of SC (−) group, there were 9 cases in which ddPCR could not be performed because of an insufficient amount of DNA collected. There was one case in which the Ex19 was detected in sputum samples in the SC (−) group. The EGFR mutation status was identical to that of FFPE sections of the tumor specimen. The sensitivity, specificity, and positive predictive value to detect EGFR mutations (Ex19 and Ex21) were 3.1%, 100%, and 100%, respectively, in SC (−) cases (Table 2).

Table 3 presents the comparison of the clinicopathological features between 13 cases of SC (+) and 105 cases of SC (−) + SNC. Compared to the SC (−) + SNC group, the CT tumor size was larger and PET SUVmax was higher in SC (+) group. Patients with SC (+) were in a more advanced stage (clinically and pathologically) compared to patients with SC (−). STAS was detected more frequently in FFPE sections of resected tumor in the SC (+) group compared to the SC (−) group (92.3% vs 34.3, p < 0.001). There was no significant difference in the EGFR mutation status between the two groups (p = 0.902); there were 61.5% and 43.8% of wild-type EGFR in SC (+) and SC (−) + SNC group, respectively.

**Table 3:** Comparison of the clinicopathological features between SC (+) and SC (−) + SNC

Total n = 118	SC (+) (n = 13)	SC (−) + SNC (n = 105)	P values^a^
Age (range)	73 (53—83)	68 (37—87)	0.711^b^
Male	7 (53.8%)	62 (59.0%)	0.711
Current or ex-smoker	11 (84.6%)	72 (68.6%)	0.340
Right side	9 (69.2%)	58 (55.2%)	0.389
*Lobe*			
Upper	4 (30.8%)	62 (59.1%)	
Middle	0	6 (5.7%)	
Lower	9 (69.2%)	37 (35.2%)	0.675
Emphysema	1 (7.7%)	11 (10.5%)	1.000
Interstitial pneumonia	1 (7.7%)	1 (1.0%)	0.209
Tumor size (mm) (range)	37 (21—98)	22 (9—54)	< 0.001^b^
PET SUVmax (range)	7.4 (3.0—19.4)	2.3 (0—30)	< 0.001^b^
*Clinical stage (8th edition)*			0.011
cStage I	6 (46.2%)	83 (79.0%)	
cStage II	4 (30.7%)	17 (16.2%)	
cStage III	3 (23.1%)	5 (4.8%)	
*Pathological stage*			
pStage 0, I	4 (30.8%)	82 (78.1%)	
pStage II	3 (23.1%)	8 (7.6%)	
pStage III	5 (38.4%)	12 (11.4%)	
pStage IV	1 (7.7%)	3 (2.9%)	0.002
Lymphatic invasion	2 (16.7%)	19 (18.3%)	1.000
Vascular invasion	6 (50.0%)	31 (29.8%)	0.191
Pleural invasion	4 (33.3%)	27 (26.0%)	0.487
Existence of STAS	12 (92.3%)	36 (34.3%)	< 0.001
*EGFR status in FFPE*			
Ex18	0	4 (3.8%)	
Ex19	2 (15.4%)	25 (23.8%)	
Ex20	0	4 (3.8%)	
Ex21	3 (23.1%)	26 (24.8%)	
Wild-type	8 (61.5%)	46 (43.8%)	0.902

PET, positron emission tomography; STAS, spread through air spaces; EGFR, epidermal growth factor receptor; FFPE, formalin-fixed paraffin-embedded; Ex18, exon 18 mutation; Ex19, exon 19 deletion mutation; Ex20, exon 20 insertion mutation; Ex21, exon 21 L858R point mutation; SC, sputum cytology; SNC, sputum not collected

^a^Fisher’s exact test

^b^Mann–Whitney U test

**Fig. 3:**
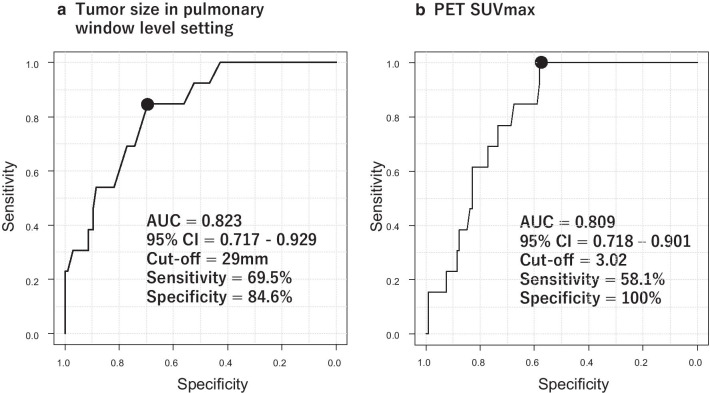
ROC curve analysis for the discrimination between SC (+) and SC (−) + SNC. The cut-off value of CT tumor size was 29 mm. The sensitivity and specificity were 69.5% and 84.6%, respectively. The cut-off value of PET SUVmax was 3.02. The sensitivity and specificity were 58.1% and 100%, respectively

Figure 3 depicts the results of ROC curve analysis for discrimination of the SC (+) and SC (−) + SNC groups. The area under the ROC curve (AUC) regarding CT tumor size was 0.823 (95% confidence interval [CI]: 0.717–0.929). The AUC regarding PET SUVmax was 0.809 (95% CI 0.718–0.901). There was no significant difference between the AUC of both groups (p = 0.809). The cut-off value of CT tumor size was 29 mm in ROC curve analysis, and the sensitivity and specificity were 69.5% and 84.6%, respectively. The cut-off value of PET SUVmax was 3.02, and the sensitivity and specificity were 58.1% and 100%, respectively.

Multivariate analysis based on a logistic regression model revealed that CT tumor size (odds ratio = 10.6, 95% CI 1.85–61.0, p = 0.008) and STAS (odds ratio = 17.7, 95% CI 1.97–158, p = 0.010) were independent potential predictive factors for SC (+) (Table 4).

**Table 4:** Logistic regression model analysis of sputum cytology positive patients

	Univariate analysis			Multivariate analysis		
	OR	95% CI	p value	OR	95% CI	p value
Age (65 ≤)	0.70	0.21–2.31	0.560			
Gender (male)	0.81	0.25–2.58	0.720			
Smoking (current or ex-smoker)	2.52	0.53–12.0	0.246			
Right side	0.55	0.56–1.89	0.342			
Lobe (upper)	3.24	0.94–11.2	0.063			
Emphysema	0.71	0.08–6.01	0.755			
Interstitial pneumonia	8.67	0.51–148	0.136			
CT tumor size (29 mm ≤)	12.5	2.63–59.9	0.002	10.6	1.85–61.0	0.008
PET SUVmax (3.1 ≤)	5.73	2.30–14.3	< 0.001	4.54	0.43–48.4	0.211
Clinical stage (II ≤)	4.23	1.73–10.3	0.002	1.47	0.31–6.91	0.627
Pathological stage (II ≤)	6.09	2.49–14.9	< 0.001	1.87	0.37–9.59	0.451
Lymphatic invasion	0.90	0.18–4.42	0.891			
Vascular invasion	2.35	0.70–7.87	0.164			
Pleural invasion	1.43	0.40–5.12	0.586			
STAS	23.0	2.87–184	0.003	17.7	1.97–158	0.010
Ex19 or Ex21mutation	0.66	0.20–2.16	0.493			

PET, positron emission tomography; SUV, standard uptake value; STAS, spread through air spaces; EGFR, epidermal growth factor receptor; Ex19, exon 19 deletion mutation; Ex21, exon 21 L858R point mutation; OR, odds ratio; CI, confidence interval

## Discussion

This is the first report of the detection of EGFR mutations of primary lung adenocarcinoma using ddPCR from prospectively collected sputum samples and compared it with an EGFR mutation in surgically resected lung cancer. EGFR mutations can be detected with high sensitivity by ddPCR from sputum sample if the sputum cytology is positive. Since a CT tumor size ≥ 29 mm is a potential predictive factor for sputum cytology positive, sputum should be collected in such cases for the EGFR mutation analysis.

Bronchoscopic, CT-guided, and surgical biopsies are currently performed in clinical practice to obtain tumor tissue for molecular analysis. However, these methodologies are invasive. An overall complication rate of 1.55% was reported for bronchoscopic biopsy, and included bleeding (0.63%) and pneumothorax (0.44%), with a mortality rate of 0.003% [8]. The reported rates of mortality and serious complications for CT-guided percutaneous needle biopsy were 0.07% and 0.75%, respectively, with complication rates of 35% for pneumothorax [9, 10]. Furthermore, surgical biopsy requires general anesthesia and is more invasive. The reported mortality rate due to surgery was 0.5%, and the rate of complications that included pneumonia, air leakage, and atelectasis was 9.6% [11]. Plasma can be collected with minimal invasion from lung cancer patients to detect EGFR mutations in cfDNA. However, there is a possibility that EGFR mutations cannot be detected in patients with EGFR mutant lung cancer if the amount of cDNA is below the threshold of detection sensitivity [12]. For this reason, liquid biopsy is performed if a tissue biopsy cannot be performed in patients with poor conditional status. Similar to plasma, sputum can be collected non-invasively, which is a greater advantage for cancer patients compared to other biopsies. The use of sputum may be another option to detect EGFR mutations in patients who are in poor condition.

Tumor cells in sputum specimens were reportedly detected in < 1% of the cells contained in sputum [15], and sputum has been considered unsuitable for molecular analysis. The present study demonstrates that ddPCR can detect EGFR mutations in primary lung adenocarcinoma with high sensitivity (80.0%) and high specificity (100%) in SC (+) cases. One discordant case in SC (+) (Case 4, invasive lepidic adenocarcinoma) was suspected to be squamous cell carcinoma (Class IIIa) according to the cytomorphologic features of sputum cytology. The discordance of EGFR mutation between sputum and surgical specimens might be due to the absence of tumor cells in the sputum. Case 4 had a history of bronchial asthma, obstructive airway disease, and lung cancer surgery. Bronchial cell hyperplasia, reactive/atypical bronchial cell, or squamous metaplasia that might be misinterpreted as malignancy, can often be observed in respiratory cytology specimens obtained from patients with asthma, chronic obstructive airway disease, inflammatory disease of the lung, and past history of lung disease chemotherapy/radiation/surgical treatment [26, 27]. When performing EGFR mutation analysis in combination with sputum cytology, it is necessary to understand the cytomorphologic features and past history of lung disease in these patients. In contrast, we observed that the sensitivity of EGFR mutation detection was as low as 3.1% in SC (−) cases, and it was considered irrelevant to perform EGFR mutation analysis unless the cytology was positive. Only one case in which the EGFR mutation (Ex19) was detected in the sputum sample in the SC (−) case. Malignant cells might not have been detected by sputum cytology. Sputum cytology involves abundant normal cells, which sometimes make it difficult to detect malignant cells. Endo et al. reported that cytotechnologists with less cytology experience under-diagnosed significantly more frequently than those with considerable experience [28]. When performing EGFR mutation testing in combination with sputum cytology, well-trained cytotechnologists are needed to make cytological diagnoses.

Hubers et al. reported that the sensitivity of EGFR mutation detection was 30 to 50% in 10 sputum samples using four different EGFR mutation analyses (Cycleave PCR, COLD-PCR, Pangaea Biotech SL Technology, and High Resolution Melting) [29]. Su et al. performed amplification refractory mutation system (ARMS)-PCR for 35 sputum samples containing tumor cells collected from stage III-IV lung cancer patients and reported a 90.9% sensitivity [30]. Wu et al. reported a 63% sensitivity when an EGFR sensitizing mutation was analyzed in 50 sputum samples using next-generation sequencing [31]. Presently, ddPCR analysis of 80 sputum samples revealed an 80.0% detection sensitivity for EGFR mutations if the sputum cytology was positive. Recently, Wang et al. reported that detection sensitivity and specificity for EGFR mutations of 46.2% and 100%, respectively, as detected using SuperARMS using sputum cell-free DNA from 102 sputum samples [32]. It is necessary to prospectively examine in large scale which methodology can detect EGFR mutations more accurately in sputum.

It is unclear which sputum collection method was appropriate for the analysis of EGFR mutations. Hubers et al. used Saccomanno's fixative (2% polyethylene glycol in 50% ethanol) for 3-day pooled sputum [29, 33]. Su et al. collected spontaneous sputum in a 1.5 mL container [30]. Wu et al. collected approximately 5 mL of spontaneous sputum in a mixed solution with an equal volume of Saccomanno's fixative and 0.005% dithiothreitol solution at a 1:1 ratio [31, 34]. We used YM fixative solution for 3-day pooled sputum. It has been reported that the detection sensitivity of lung cancer in sputum is increased by a longer duration of sputum collection and with the induction of sputum by nebulization with hypertonic saline [33, 35]. A future study should examine whether the sensitivity of EGFR mutation detection can be improved by different sputum collection methodologies.

This study showed that EGFR mutation analysis of sputum should be performed for SC (+). However, little is known about the risk factors for SC (+). Therefore, sub-analysis of risk factors for SC (+) in patients with primary lung adenocarcinoma was performed in this study. As shown in Fig. 3 and Table 4, we found that CT tumor size was strongly associated with SC (+). Patients with CT tumors ≥ 29 mm were considered good candidates for EGFR mutation analysis. The detection sensitivity of lung cancer in sputum cytology was reported to be 40 to 66% [13, 14]. Presently, the sensitivity was 11.0%. There are two reasons of the low sensitivity we observed. Firstly, all the patients had lung adenocarcinoma. Sputum cytology is highly effective for central type squamous cell carcinoma in patients with hemoptysis [16]. Sing et al. reported that the detection rate of sputum in adenocarcinoma in 64 patients was 25.0% [14]. Secondly, patients with early staged lung cancer were included. The sensitivity of sputum cytology for patients with advanced stages are reported to be higher than for early stage cancer [14]. The diagnostic sensitivity of bronchoscopic biopsy was reported to be 88% (78% for peripheral lung cancer) [36]. The sensitivity of percutaneous needle biopsy was 86.1% [37]. Although sputum can be collected non-invasively from lung cancer patients, the detection sensitivity of lung cancer was lower compared to other methodologies. In sub-analysis of this study revealed that the sensitivity increased to 69.5% in sputum cytology if the CT tumor size was ≥ 29 mm. Risse et al. similarly reported that the detection sensitivity of primary lung cancer was high when the tumor size exceeded 24 mm [16].

This is the first study to analyze the correlation between pathological findings of surgically resected specimens and SC (+). SC (+) status was strongly associated with STAS. The presence of STAS was higher in patients with SC (+) than in patients with SC (−) (92.3% vs. 34.3%). STAS was a potential predictive factor for SC (+) in multivariate analysis (Table 4). STAS is a risk factor for recurrence of primary lung adenocarcinoma and squamous cell carcinoma [38, 39]. Previously, we examined the morphology and EGFR mutation status of tumor cells in airway secretions collected from segmental or lobar bronchus of surgically resected specimens and compared the results with FFPE tumor tissue. The study demonstrated that STAS may be spread to the respiratory tract as far as segmental or lobar bronchus of the tumor [40]. Because STAS was a risk factor for SC (+) in the present study, we suggest that malignant tumors can be efficiently detected from sputum if STAS is predicted in preoperative radiological findings. Toyokawa et al. reported that the presence of notch and the absence of ground glass opacity were CT findings that were related to the presence of STAS [41]. Kim et al. reported that solid component ratio ≥ 90% in CT was a potential predictive factor of STAS [42]. Performing sputum cytology for tumors that display these CT findings may increase the detection sensitivity of sputum cytology in patients with primary lung cancer.

There were several limitations in our study. First, this study was conducted at a single institution with a small number of patients. Second, we have not investigated the efficacy of EGFR-tyrosine kinase inhibitor based on the EGFR status detected in sputum samples. Third, the methodology of detecting EGFR mutation differed between surgical resected tumor samples and sputum samples. The discordance of EGFR mutations between the two samples might have occurred if the number of tumor cells harboring EGFR mutations with mutations was too small to detect these mutations, due to heterogeneity in FFPE tumor sections by conventional PCR. Fourth, it is unclear whether performing EGFR mutation testing of sputum based on sputum cytology results is valid. Further studies are needed to investigate the clinicopathological factors of lung cancer patients who are clinically relevant for sputum-based EGFR mutation testing. Fifth, this study examined only the Ex21 and Ex19. Future studies will need to detect other EGFR mutations, such as T790M, and other driver gene mutations by ddPCR from sputum, and evaluate the usefulness of ddPCR for sputum in clinical practice. Detection of T790M for the indication of Osimertinib is especially important in clinical practice. The detection of T790M from sputum may play an important role similar to that of cell-free DNA in plasma. Further studies are needed to demonstrate the clinical usefulness of T790M detection in sputum using ddPCR in patients with primary lung cancer undergoing EGFR-TKI treatment. Further research also is necessary to compare the detection rates of EGFR mutations between sputum and cell-free DNA in plasma.

## Data Availability

The datasets used and/or analysed during the current study are available from the corresponding author on reasonable request.

## Abbreviations

NSCLC: Non-small cell lung cancer
EGFR: Epidermal growth factor receptor
Ex21: EGFR exon 21 L858R point mutation
Ex19: EGFR exon 19 deletion mutation
CT: Computed tomography
ddPCR: Droplet digital polymerase chain reaction
SC (+): Sputum cytology positive
SC (−): Sputum cytology negative
SNC: Sputum not collected
H&E: Hematoxylin and eosin
FFPE: Formalin-fixed paraffin-embedded
STAS: Spread through air space
ROC: Receiver operating characteristics
PET SUVmax: Positron emission tomography maximum standardized uptake value
18F-FDG: Fluorodeoxyglucose F 18
AUC: Area under curve
